# Clinical utility of point-of-care lactate measurement for assessing management status and prognosis in dogs with myxomatous mitral valve disease

**DOI:** 10.3389/fvets.2026.1759873

**Published:** 2026-02-20

**Authors:** Wangyu Choi, Ji-young Ha, Eon-Seung Jeong, Young-Min Yun

**Affiliations:** 1 College of Veterinary Medicine, Jeju National University, Jeju-si, Republic of Korea; 2 Jamsil Best Animal Medical Center, Seoul, Republic of Korea; 3 SEEU Animal Medical Center, Seoul, Republic of Korea

**Keywords:** dogs, hemodynamic instability, Lactate, myxomatous mitral valve disease, point-of-care testing, veterinary internal medicine, prognosis

## Abstract

**Introduction:**

Myxomatous mitral valve disease (MMVD) is a chronic cardiac condition that can have serious hemodynamic consequences and therefore requires lifelong monitoring. However, although echocardiography remains the gold standard for diagnosis and staging, its repetition at short intervals to assess therapeutic response can be limited by cost, logistics, and patient tolerance. In this context, biomarkers such as lactate could serve as a complementary tool to reflect clinical status and prognosis. This study aimed to evaluate the clinical utility of point-of-care (POC) whole blood lactate measurement in dogs with MMVD, with a particular focus on its ability to reflect clinical status within American College of Veterinary Internal Medicine (ACVIM) stages B2 and C.

**Methods:**

This retrospective observational study included 33 client-owned dogs with MMVD examined between June 2025 and November 2025. The population had a mean age of 13.6 years and mean body weight of 4.3 kg. Dogs were classified according to ACVIM stage and clinical status at presentation into three groups: Stage B2 (*n*=13), Stage C with controlled congestive heart failure (CHF) (*n*=13), and Stage C with acute decompensated CHF (*n*=7). Whole blood lactate concentrations were measured using a portable lactate meter (StatStrip Xpress^®^) via paw pad capillary sampling within approximately 5-minute of acclimatization.

**Results:**

Whole blood lactate concentrations differed significantly among the three groups (*P* = 0.002). The Stage C with acute decompensation CHF group exhibited a markedly higher mean lactate concentration (5.61 ± 1.98 mmol/L) compared to the Stage B2 (2.17 ± 0.95 mmol/L) and Stage C with controlled CHF (2.50 ± 1.04 mmol/L) group (*P* = 0.002). In contrast, no significant difference was observed between the Stage B2 and Stage C with controlled CHF groups (*P* = 0.456). In receiver operating characteristic (ROC) analysis for distinguishing dogs with hemodynamic instability, the area under the curve (AUC) was 0.94 (95% CI: 0.85–1.00, *P* < 0.001). At a cut-off value of 3.0 mmol/L, the assay demonstrated 100% sensitivity, 72% specificity, and a 100% negative predictive value (NPV). Lactate concentrations ≥ 3.0 mmol/L were observed in 2 of 13 dogs (15.4%) in the Stage B2 group, 3 of 13 dogs (23.1%) in the Stage C with controlled CHF group, and 7 of 7 dogs (100%) in the Stage C with acute decompensated CHF group.

**Discussion:**

POC whole blood lactate measurement can serve as a valuable adjunctive screening tool for assessing clinical management status and acute hemodynamic condition in dogs with MMVD. However, lactate values approximating the identified cut-off of 3.0 mmol/L should not be interpreted as a binary decision threshold. Instead, values in this range indicate a zone of increased clinical uncertainty, warranting closer monitoring and hemodynamic reassessment in conjunction with standard clinical evaluation.

## 1 Introduction

Myxomatous mitral valve disease (MMVD) is the most common acquired heart disease in dogs, accounting for approximately 75% of canine heart diseases (1, 2). The disease is characterized by progressive degeneration of the mitral valve, leading to mitral regurgitation, volume overload, and ultimately congestive heart failure (CHF) (1). Mitral regurgitation results in reduced effective forward stroke volume, while chronic volume overload leads to progressive ventricular remodeling and increased myocardial wall stress. Together, these changes can impair cardiac output and contribute to hemodynamic instability, particularly during episodes of acute decompensation. According to the American College of Veterinary Internal Medicine (ACVIM) consensus guidelines, MMVD is classified into four stages (A to D) based on structural remodeling and the presence of clinical signs (3). In particular, the transition from the asymptomatic Stage B2 to the symptomatic Stage C with overt congestive heart failure represents a pivotal turning point for therapeutic intervention and prognosis. The Evaluation of Pimobendan In dogs with Cardiomegaly (EPIC) trial (4) demonstrated that initiating pimobendan at the B2 stage significantly delays the onset of overt heart failure and prolongs survival, underscoring the clinical importance of accurately identifying and monitoring dogs at this stage.

Currently, the gold standard for staging and monitoring MMVD involves a combination of echocardiography, thoracic radiography, and cardiac biomarkers such as N-terminal pro-B-type natriuretic peptide (NT-proBNP) (3, 5, 6). Echocardiography provides essential structural and hemodynamic information and remains central to diagnosis and follow-up. In clinically stable dogs with well-controlled CHF, echocardiographic reassessment at intervals of several months is generally sufficient, as recommended by current guidelines (3). However, during periods of treatment initiation, medication adjustment, suspected clinical deterioration, or uncertainty regarding therapeutic response or owner compliance, more frequent clinical reassessment is often required. In such situations, performing comprehensive echocardiography at every visit may be impractical due to patient tolerance, logistical constraints, and cost, particularly in dogs presenting with respiratory distress or acute clinical instability. As a practical alternative, Point-of-Care Ultrasound (POCUS) can facilitate rapid bedside assessment of cardiac size, pulmonary edema, or pleural effusion (7, 8) These ultrasonographic findings, however, primarily reflect structural or compartmental changes and are not designed to directly quantify the overall adequacy of systemic tissue perfusion at the time of presentation.

Moreover, physical restraint and the hospital environment may provoke stress-related physiological responses, potentially influencing heart rate, blood pressure, and respiratory patterns in dogs with cardiorespiratory compromise (9). Clinical evaluation of dogs with suspected acute decompensation therefore relies on integrated interpretation of physical examination findings, imaging results, and laboratory data. In this context, blood-based markers are well suited to reflect the global circulatory and metabolic consequences of acute hemodynamic compromise, complementing structural assessment of the heart and thorax.

Within this framework, blood-based biomarkers are routinely incorporated into the clinical evaluation of dogs with MMVD. While established cardiac biomarkers such as NT-proBNP are primarily used to support diagnosis and staging (6), other circulating markers have been explored to provide complementary information regarding myocardial injury, extracardiac organ involvement, and systemic consequences of the disease.

Various circulating biomarkers have been investigated to support disease monitoring and early detection of clinical deterioration in dogs with MMVD. Cardiac troponin I reflects myocardial injury and disease severity, while symmetric dimethylarginine (SDMA) has been proposed as a marker of renal function that may aid risk stratification in cardiac patients receiving chronic diuretic therapy (10, 11). These biomarkers are clinically valuable for characterizing myocardial injury and comorbid organ involvement; however, they do not directly quantify global tissue perfusion and may be less responsive to rapid, short-term fluctuations in circulatory status at the time of presentation. Accordingly, a rapid, minimally stressful point-of-care measurement of blood lactate can serve as a practical adjunctive indicator of circulatory adequacy, particularly during episodes of suspected acute clinical deterioration. Importantly, as with other biomarkers, lactate values should be interpreted as one component of a comprehensive clinical assessment rather than as a standalone diagnostic tool.

Blood lactate is a well-established metabolic marker of tissue hypoperfusion and anaerobic metabolism (12). In veterinary emergency medicine, hyperlactatemia is widely used to stratify risk and predict outcome in conditions such as gastric dilatation-volvulus (GDV), sepsis, and shock (12, 13). In the context of MMVD, Silva-Filho et al. (14) demonstrated that venous lactate concentrations increase progressively with advancing ACVIM stages (B1–D) and proposed a lactate cut-off value of 3.35 mmol/L to distinguish dogs with cardiac remodeling from healthy controls, suggesting that high lactate levels are associated with an increased risk of progression to overt heart failure. More recently, Petchdee et al. (15) evaluated right ventricular function, echocardiographic indices, and serum peptidomic profiles in 93 dogs with MMVD. They reported significantly higher lactate levels in Stage C compared to Stage B and identified lactate, along with vertebral heart score (VHS) and left atrial-to-aortic root ratio (LA/Ao ratio), as a potential prognostic indicator for cardiac death. However, both studies relied on central laboratory analysis of venous blood and primarily focused on the anatomical disease stage or long-term prognosis. Furthermore, these studies did not explicitly differentiate patients based on their hemodynamic stability or therapeutic response within Stage C.

Several previous studies have acknowledged the marked clinical heterogeneity of dogs classified as ACVIM Stage C and have used descriptive or conceptual approaches to characterize differences in clinical presentation, disease severity, or short-term risk, without proposing formal modifications to the ACVIM staging system (16). In particular, investigations evaluating dogs with MMVD and congestive heart failure have demonstrated that hematological, biochemical, and echocardiographic variables are associated with survival, thereby reinforcing the substantial variability in clinical status and prognosis among dogs within the same stage (17).

In clinical practice, dogs classified as ACVIM Stage C represent a clinically heterogeneous population. According to current consensus guidelines and standard internal medicine references, Stage C includes dogs with a history of congestive heart failure that may be clinically controlled at the time of evaluation as well as dogs presenting with active or severe congestive signs requiring urgent management. According to standard internal medicine references, dogs classified as ACVIM Stage C are defined by the presence of current or previous clinical signs of congestive heart failure, with clinical status at presentation varying from controlled CHF to acute decompensated CHF (18). As a result, dogs within Stage C may present with differing degrees of hemodynamic compromise at the time of evaluation. Consequently, grouping all Stage C patients into a single category may obscure clinically relevant differences in circulatory status at the time of evaluation. In this context, identifying patients presenting with acute decompensation is particularly important, as these dogs exhibit impaired tissue perfusion and physiological compromise.

From a clinical perspective, assessment of systemic perfusion becomes relevant when evaluating dogs with advanced MMVD presenting with varying clinical severity. Assessing lactate in these patients, however, presents a practical challenge. Nevertheless, given the marked clinical heterogeneity within ACVIM Stage C, there is a need for a readily accessible indicator that can further characterize differences in circulatory status at the time of presentation. While jugular venipuncture is the standard method for obtaining blood samples, the physical restraint required can be life-threatening for dyspneic dogs with acute decompensated heart failure. To address this issue, we utilized handheld lactate meters, which require only a minimal blood volume and offer results within seconds, enabling minimally invasive monitoring via paw pad capillary sampling (19–22). This approach reduces patient stress and restraint, making it a practical alternative for critically ill patients who cannot tolerate standard venipuncture.

This retrospective observational study aims to investigate the relationship between whole blood lactate concentrations and the clinical management status of dogs with MMVD. We specifically assessed whether lactate levels differ between with Stage C with controlled CHF and those presenting with Stage C with acute decompensated CHF, rather than considering Stage C as a single homogeneous group. In addition, lactate concentrations in dogs with Stage C with controlled CHF were compared with those in asymptomatic Stage B2 dogs. Finally, this study explored the potential clinical relevance of lactate measurement as an adjunctive indicator of acute clinical deterioration in dogs with MMVD.

## 2 Materials and methods

### 2.1 Study design

This was a retrospective, clinical, observational study conducted at two veterinary referral centers (Jamsil Best Animal Medical Center and SEEU Animal Medical Center). Medical records of dogs diagnosed with MMVD between June 2025 and November 2025 were reviewed.

### 2.2 Ethics statement

The study protocol was approved by the Institutional Animal Care and Use Committee (IACUC) of Jeju National University (Approval No. 2025–0105).

### 2.3 Animals and experimental design

The study included 33 client-owned dogs diagnosed with myxomatous mitral valve disease (MMVD) according to the ACVIM consensus guidelines (3). All dogs were staged based on ACVIM criteria at the time of enrollment. As this analysis was retrospective, the sample size was determined by case availability rather than by prospective power calculation.

Dogs classified as Stage B2 (*n* = 13) were asymptomatic for congestive heart failure (CHF) but had evidence of significant cardiac remodeling, defined by a left atrial-to-aortic root ratio (LA/Ao) ≥1.6 and a vertebral heart score (VHS) >10.5. Dogs classified as Stage C (*n* = 20) had current or previous clinical signs consistent with CHF and were receiving medical therapy for heart failure. Stage D represents the refractory phase of the disease, characterized by persistent heart failure despite standard doses of recommended cardiac medications and the requirement for furosemide at daily doses exceeding 8 mg per kilogram of body weight.

**Figure 1:**
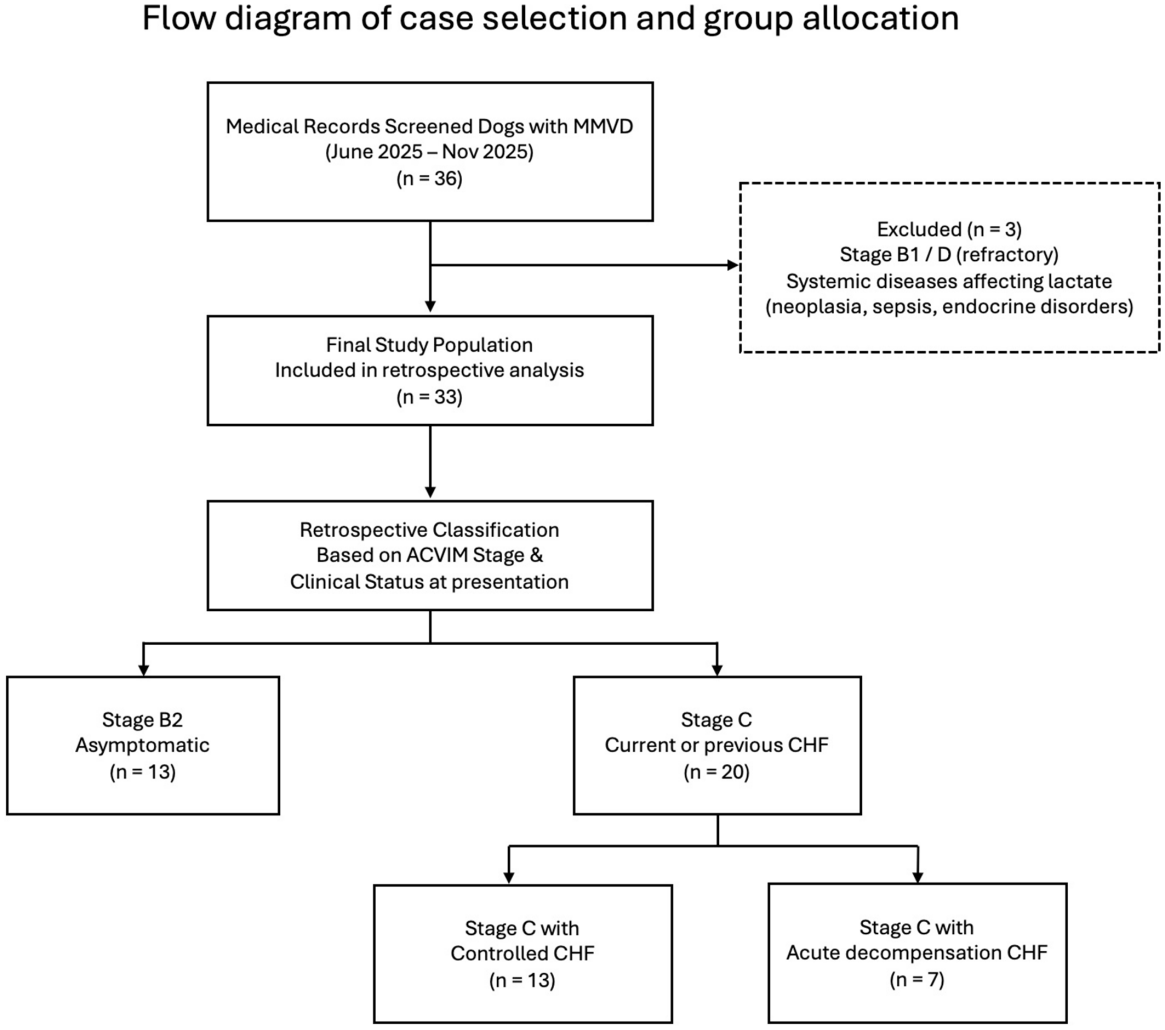
Flow diagram illustrating the retrospective screening of medical records of dogs diagnosed with MMVD between June 2025 and November 2025 at two veterinary referral centers. Dogs were excluded based on predefined criteria, including refractory disease stage or systemic conditions known to affect blood lactate concentrations. The final study population was retrospectively classified according to ACVIM stage and clinical status at presentation into Stage B2 and Stage C groups. Dogs classified as Stage C were further stratified into clinically controlled CHF and acute decompensation CHF at the time of evaluation.

The process of case selection, exclusion criteria, and final group allocation is summarized in Figure 1. Dogs classified as Stage D were excluded from the study because this stage reflects refractory heart failure requiring advanced or rescue therapeutic strategies, which was beyond the scope of the present investigation. Accordingly, ACVIM staging in this study was determined based on clinical status and treatment requirements at the time of presentation.

Within the ACVIM staging framework, dogs classified as Stage C explicitly include those with either first-onset or recurrent congestive heart failure and encompass patients evaluated both during acute presentations requiring emergency management and during periods of clinical stability. Given that this definition inherently reflects a clinically heterogeneous population, dogs in this study were further stratified according to their clinical status at the time of presentation to allow for a more refined clinical characterization. The Stage C–acute decompensation group (*n* = 7) comprised dogs presenting with acute worsening of congestive heart failure, as determined by the attending clinician based on clinical signs and the need for immediate therapeutic intervention at the time of evaluation.

Medication history, including documented nonadherence or withdrawal prior to presentation, was recorded when available in the medical records but was not used as a primary criterion for subgroup classification. The following variables were collected for each dog: signalment (age and body weight), ACVIM stage as defined by current consensus guidelines, clinical status at presentation, whole blood lactate concentration, and relevant echocardiographic indices used for staging. Dogs with systemic diseases known to affect blood lactate concentrations, such as neoplasia, sepsis, or unmanaged endocrine disorders, were excluded.

### 2.4 Sample collection and lactate measurement

To minimize the influence of post-prandial metabolism, owners were instructed to withhold food for at least 4 h prior to the visit whenever possible. For emergency cases presenting with acute clinical signs, lactate measurement was performed regardless of feeding status, as a previous study in healthy dogs demonstrated that post-prandial lactate elevation is minimal and remains within the clinical reference interval (23). Feeding status at the time of sampling was recorded and considered during data interpretation; however, given the reported minimal post-prandial effect on lactate concentration, this variability was not expected to substantially confound the assessment of acute hemodynamic status in emergency settings. Upon arrival, whole blood lactate concentrations were measured within approximately 5-min in a quiet environment, without a prolonged acclimatization period, reflecting routine clinical practice. During lactate measurement, care was taken to minimize stress and physical restraint in order to reduce the potential influence of stress-related hyperlactatemia (9).

Whole blood lactate concentrations were measured using a portable lactate meter (StatStrip Xpress® Lactate Meter, Nova Biomedical, Waltham, MA, USA). To minimize patient handling, a capillary blood sample (0.3–0.6 μL) was obtained *via* a minimally invasive paw pad puncture using a sterile lancet after cleaning the site.

### 2.5 Statistical analysis

Statistical analyses were performed using GraphPad Prism 9.0 (GraphPad Software, San Diego, CA, USA). The normality of data distribution was evaluated using the Shapiro–Wilk test. Differences in lactate concentrations among the three groups were analyzed using the Kruskal-Wallis test, followed by Dunn’s post-hoc test for multiple comparisons. Demographic variables including age and body weight were compared using the Kruskal-Wallis test, and sex distribution was analyzed using Fisher’s exact test.

The diagnostic performance of lactate for predicting hemodynamic instability was evaluated using Receiver Operating Characteristic (ROC) curve analysis. The optimal cut-off value was determined using the Youden index, and the sensitivity, specificity, positive predictive value, and negative predictive value were calculated. A *p*-value < 0.05 was considered statistically significant.

A post-hoc power calculation determined that the sample size provided a statistical power of 96% (power = 0.96, *α* = 0.05) to detect significant differences in lactate concentrations between the Stage C controlled and acute decompensated groups, given the large effect size observed (Cohen’s *d* = 2.01).

## 3 Results

### 3.1 Demographic characteristics

Table 1 summarizes the demographic and clinical characteristics of the study population. A total of 33 dogs with MMVD were included in the final analysis. The dogs were classified into three groups based on their clinical status: Stage B2 (*n* = 13), Stage C with controlled CHF (*n* = 13), and Stage C with acute decompensation CHF (*n* = 7). The study population was well-balanced across the groups. There were no statistically significant differences in age (*p* = 0.582), body weight (*p* = 0.865), sex (*p* = 0.841), or breed distribution (*p* = 0.652) among the three groups, ensuring that baseline characteristics did not confound the lactate comparison.

**Table 1:** Demographic and clinical characteristics of the study population.

Variable	Stage B2 (*n* = 13)	Stage C with controlled CHF (*n* = 13)	Stage C with acute decompensation CHF (*n* = 7)	*p*-value
Age (years)	13.5 ± 3.4	12.5 ± 3.0	14.0 ± 2.3	0.582
Body weight (kg)	4.2 ± 2.2	3.8 ± 1.2	4.4 ± 1.3	0.865
Sex (male/female)	6/7	6/7	2/5	0.841
Breed, n (maltese/poodle/others)	4/4/5	7/2/4	5/1/1	0.652
Lactate (mmol/L) (mean ± SD)	2.17 ± 0.95	2.50 ± 1.04	5.61 ± 1.98	<0.002*

Data are presented as mean ± standard deviation or number. Others include Pomeranian (*n* = 3), Mixed breed (*n* = 2), Chihuahua (*n* = 1), Japanese Spitz (*n* = 1), Cavalier King Charles Spaniel (*n* = 1), Cocker Spaniel (*n* = 1), and Schnauzer (*n* = 1).

### 3.2 Comparison of whole blood lactate concentrations between groups

**Figure 2:**
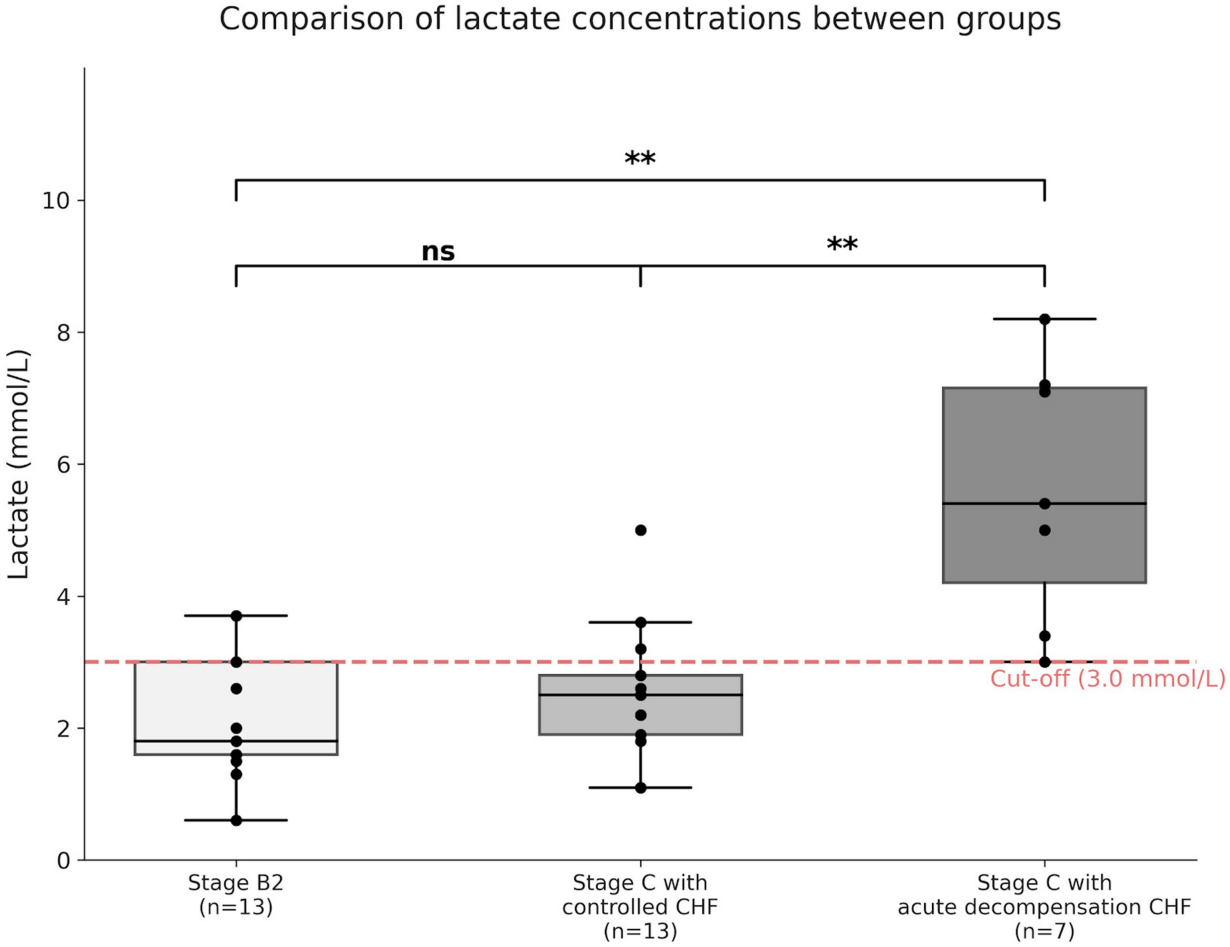
Comparison of whole blood lactate concentrations among clinical groups. The red dashed horizontal line indicates the ROC-derived cut-off value (3.0 mmol/L). Dogs with stage C with acute decompensated CHF showed significantly higher lactate concentrations compared with dogs in stage B2 (*p* = 0.002, Dunn’s *post hoc* test) and stage C with controlled CHF (*p* = 0.002, Dunn’s post hoc test). No significant difference was observed between the stage B2 and stage C with controlled CHF groups (*p* = 0.456). The boxes represent the interquartile range (IQR), the horizontal line inside within each box indicates the median, and the whiskers extend to the minimum and maximum values. Individual data points are shown as black dots.

The distribution of whole blood lactate concentrations across the three groups is presented in Figure 2 and Table 2. The lactate concentrations ranged from 0.6 to 8.2 mmol/L across the entire study population. A statistically significant difference in lactate concentrations was observed among the three groups (Kruskal-Wallis test, *p* = 0.002; Figure 2). Post-hoc analysis revealed that the Stage C with acute decompensation CHF group had a significantly higher mean lactate concentration (5.61 ± 1.98 mmol/L) compared to both the Stage B2 group (2.17 ± 0.95 mmol/L, *p* = 0.002) and the Stage C with controlled CHF group (2.50 ± 1.04 mmol/L, *p* = 0.002). In contrast, no statistically significant difference was observed between the Stage B2 group and the Stage C with controlled CHF group (*p* = 0.456). When evaluating the distribution based on the cut-off value derived from ROC analysis, Lactate concentrations ≥ 3.0 mmol/L were observed in 2 of 13 dogs (15.4%) in the Stage B2 group, 3 of 13 dogs (23.1%) in the Stage C with controlled CHF group, and 7 of 7 dogs (100%) in the Stage C with acute decompensated CHF group (Figure 2; Table 2).

**Table 2:** Detailed distribution of whole blood lactate concentrations across clinical groups.

Group	*N*	Mean ± SD (mmol/L)	Median (mmol/L)	Range (min—max)
Stage B2	13	2.17 ± 0.95	1.8	0.6–3.7
Stage C with controlled CHF	13	2.50 ± 1.04	2.5	1.1–5.0
Stage C with acute decompensation CHF	7	5.61 ± 1.98	5.4	3.0–8.2

Lactate values are summarized for each group using measures of central tendency and dispersion, including mean with standard deviation, median, and observed range. The distribution demonstrates substantial overlap in lactate concentrations between dogs with Stage B2 and those with Stage C and controlled congestive heart failure, whereas dogs with Stage C and acute decompensated congestive heart failure show a clear upward shift in both central tendency and overall range.

### 3.3 Diagnostic performance for predicting hemodynamic instability

**Figure 3:**
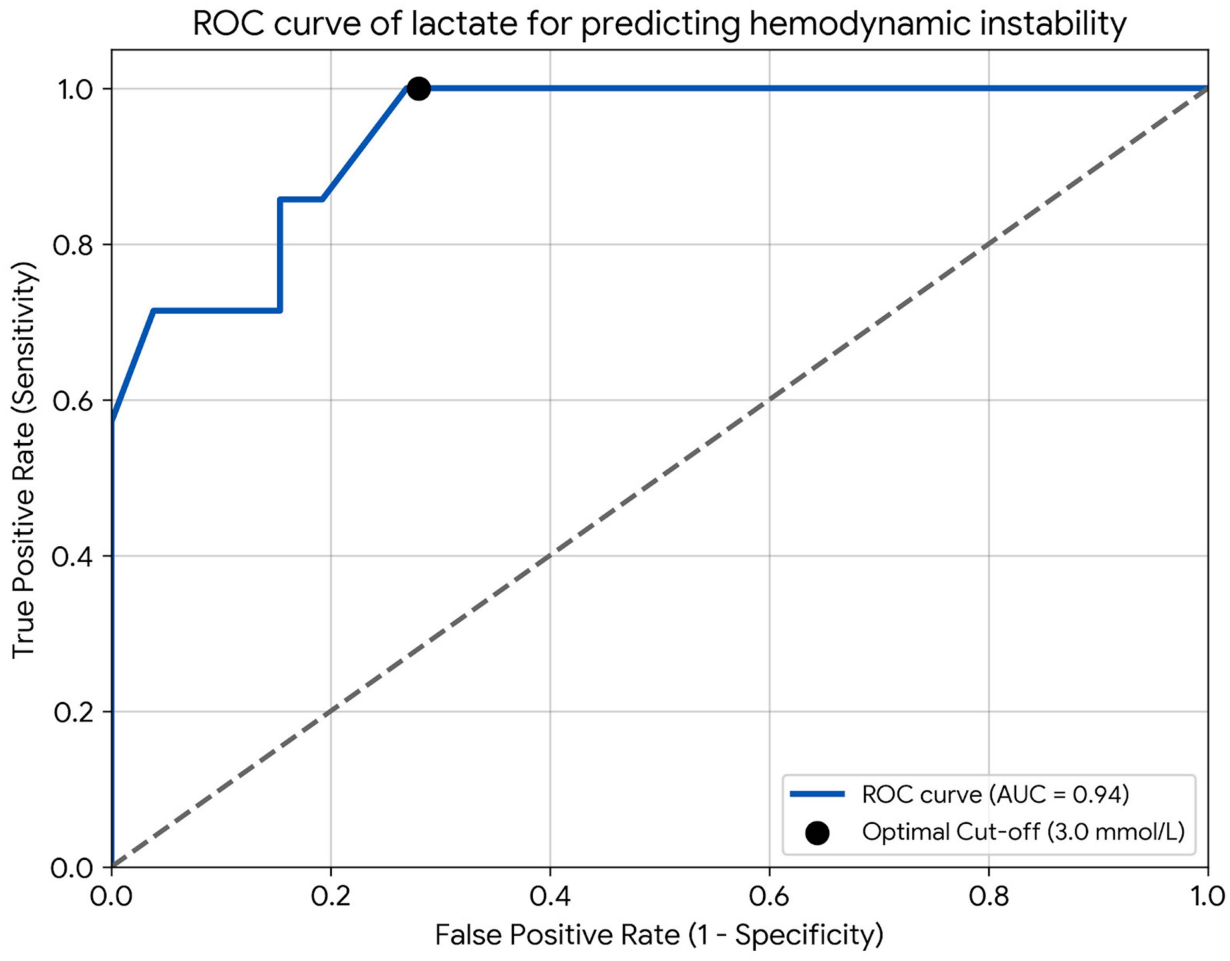
ROC curve of lactate for predicting hemodynamic instability. The area under the curve (AUC) was 0.94 (95% CI 0.85–1.00, *p* < 0.001), indicating high diagnostic accuracy. The black dot represents the optimal cut-off value of 3.0 mmol/L, which provided a sensitivity of 100% and a specificity of 72%.

The diagnostic performance of whole blood lactate measurement for predicting hemodynamic instability is illustrated in Figure 3 and detailed in Table 3. ROC curve analysis yielded an Area Under the Curve (AUC) of 0.94 (95% CI: 0.85–1.00, *p* < 0.001), indicating high diagnostic accuracy. The optimal cut-off value determined by the Youden index was 3.0 mmol/L. At this threshold, the assay demonstrated a sensitivity of 100% and a specificity of 72.0%. Furthermore, the negative predictive value (NPV) was 100.0%.

**Table 3:** Diagnostic performance of point-of-care lactate measurement for detecting hemodynamic instability (Cut-off value: 3.0 mmol/L).

Lactate level	Stage C with acute decompensation CHF (actual positive)	Stage B + stage C with controlled CHF (actual negative)	Total
High (>3.0 mmol/L)	7 (TP)	7 (FP)	14
Low (<3.0 mmol/L)	0 (FN)	19 (TN)	19
Total	7	26	33

This table presents the distribution of dogs classified according to lactate concentration using a cut-off value of 3.0 mmol/L, with dogs presenting with Stage C and acute decompensated CHF considered as the target condition. The table illustrates the number of true positive, false positive, true negative, and false negative results across groups, providing the basis for calculating sensitivity, specificity, and predictive values.

## 4 Discussion

This study evaluated whole blood lactate concentrations in dogs with MMVD using a portable lactate meter with the primary aim of correlating lactate levels with clinical stability under ongoing management. A notable finding of this study is that lactate concentrations in the Stage C with controlled CHF group were indistinguishable from those in the asymptomatic Stage B2 group. Despite the advanced anatomical severity of Stage C, the mean lactate level in the Stage C with controlled CHF group was 2.50 mmol/L. This value falls within the reference interval for healthy dogs reported by Hughes et al. (24), and is consistent with previous findings showing higher lactate concentrations in dogs with Stage C MMVD compared with Stage B2, while reported values remained within a relatively narrow range in the absence of overt hypoperfusion, as reported by Soares et al. (25). In contrast, in the present study, these findings show that dogs with well-controlled CHF had lactate concentrations similar to those of asymptomatic Stage B2 dogs.

In this study, a Stage C diagnosis alone was not associated with increased lactate concentration, and lactate values remained within the normal range in the Stage C with controlled CHF group. In this context, lactate measurement can provide supportive information regarding overall metabolic stability and adequacy of clinical management. From a biological perspective, this finding may reflect preserved systemic hemodynamics under effective medical management, whereas acute decompensation is associated with transient reductions in cardiac output and impaired oxygen delivery, resulting in increased anaerobic metabolism and lactate production.

Unlike established cardiac biomarkers such as NT-proBNP and cardiac troponin I, which primarily reflect myocardial wall stress and cardiomyocyte injury, respectively (5, 11), blood lactate concentration represents a global marker of systemic hypoperfusion and metabolic stress (26). NT-proBNP is closely associated with chronic volume and pressure overload and is therefore useful for staging disease severity and monitoring long-term cardiac remodeling, whereas troponin I reflects myocardial injury and has been linked to adverse outcomes in dogs with advanced heart disease. In contrast, lactate elevation is typically driven by acute reductions in oxygen delivery and impaired tissue perfusion, providing complementary information that reflects the dynamic clinical state rather than structural disease burden alone. From this perspective, lactate measurement does not replace established cardiac biomarkers but can provide complementary, time-sensitive insight into acute hemodynamic compromise in dogs with MMVD.

The distribution of lactate concentrations differed markedly within Stage C. Dogs classified as Stage C with acute decompensated CHF exhibited higher lactate concentrations than both Stage B2 dogs and those with Stage C with controlled CHF, whereas lactate values in the Stage C with controlled CHF group overlapped substantially with those observed in Stage B2.

Taken together, our results suggest that point-of-care lactate measurement serves as a valuable real-time indicator of clinical management status in dogs with MMVD. Elevated lactate concentrations should therefore prompt careful clinical reassessment, as they are associated with inadequate therapeutic control or disease destabilization, rather than being interpreted as a standalone diagnostic marker, in line with previous critical care literature describing lactate as a clinically useful indicator of illness severity and systemic compromise in small animals (26).

Previous studies have reported an association between MMVD progression and increased blood lactate concentrations; however, important aspects of patient condition at the time of measurement and treatment context were not fully addressed. Our study builds on these findings by providing additional clinical context regarding the conditions under which lactate elevation occurs. First, Silva-Filho et al. (14) reported a progressive increase in venous lactate concentrations with advancing ACVIM stages and proposed a cut-off value of 3.35 mmol/L to distinguish dogs with cardiac remodeling from healthy controls. While their findings highlighted the relationship between anatomical severity and lactate, patients were not stratified according to hemodynamic status or response to ongoing therapy. Consequently, their results implied that advanced stage inherently leads to hyperlactatemia. In contrast, our study refines this perspective by demonstrating that even Stage C patients can maintain normal lactate levels with a mean of 2.50 mmol/L if they are hemodynamically stable. This indicates that lactate elevation is more reflective of acute decompensation rather than the chronic disease stage itself. Second, Petchdee et al. (15) identified lactate as a prognostic factor for cardiac death alongside echocardiographic indices and serum peptidomics. They reported significantly higher lactate levels in Stage C compared to Stage B. However, their analysis compared disease stages without accounting for differences in contemporaneous clinical condition, leaving uncertainty as to whether the elevated lactate reflected disease stage itself or a subset of patients experiencing acute decompensation within Stage C. Our results clarify this distinction by demonstrating that marked hyperlactatemia was confined to dogs with Stage C and acute decompensated CHF, whereas lactate concentrations remained within the normal range in dogs with controlled CHF.

Collectively, our results provide a more clinically nuanced insight that blood lactate is a dynamic biomarker that reflects real-time physiological compromise rather than static disease classification. Accordingly, monitoring lactate levels offers a practical adjunct for identifying therapeutic failure or acute decompensation in dogs with MMVD.

Beyond differentiating clinical status at presentation, our findings suggest that blood lactate concentration has potential prognostic relevance in dogs with MMVD. In this study, a lactate value around 3.0 mmol/L was observed to differ between dogs with acute clinical deterioration and those with controlled disease; however, this threshold should be interpreted with caution due to overlap between groups and the limited sample size. The limited number of dogs in the acute decompensation group, together with the observation that several individuals were located near the proposed cut-off, indicates that this value does not represent a strict boundary between clinical states but rather a continuum of physiological response.

These findings are consistent with established principles in veterinary emergency medicine, where hyperlactatemia has been associated with impaired tissue perfusion and adverse outcomes in critical conditions such as gastric dilatation–volvulus, shock, and sepsis (12, 27–31), and where lactate concentration has been shown to correlate with mortality risk in critically ill small animals (32). While these associations cannot be directly extrapolated to MMVD, the observation that the two non-surviving dogs in the present study presented with the highest lactate concentrations, reaching 5.4 mmol/L and 8.2 mmol/L, is consistent with a possible association between marked hyperlactatemia and severe clinical compromise. Moreover, previous studies have demonstrated that persistently elevated lactate concentrations or failure of lactate normalization over time are associated with poor outcomes in critically ill dogs (33). Nevertheless, the small number of non-survivors and the overlap of lactate values near the proposed cut-off suggests possible prognostic relevance, but insufficient to determine prognostic utility or validated cut-offs.

Accordingly, lactate concentrations exceeding approximately 3.0 mmol/L should not be interpreted in isolation or used as a dichotomous decision threshold. Instead, values within this range may indicate a zone of increased clinical uncertainty, in which closer monitoring and reassessment of hemodynamic status are warranted in conjunction with standard clinical and echocardiographic evaluation, particularly during episodes of acute clinical deterioration in dogs with MMVD.

Given the critical need to identify these high-risk patients rapidly, the practicality of the measurement method becomes paramount. While central laboratory analyzers remain the gold standard for lactate measurement, their requirement for venipuncture and processing time limits their utility in critical emergency scenarios (19). In dogs with Stage C MMVD presenting with acute respiratory distress, tachypnea, or severe dyspnea, the physical restraint necessary for jugular venipuncture may exacerbate clinical signs and increase the risk of further deterioration. In such settings, rapid bedside assessment that minimizes handling is clinically desirable.

The StatStrip Xpress® lactate meter has been validated in human medicine, showing strong correlation with reference methods (34). In veterinary medicine, several handheld lactate analyzers have demonstrated acceptable agreement with laboratory analyzers and clinical utility in dogs (12–15). Leveraging this reliability, we demonstrated that capillary blood sampling from the paw pad allows for rapid measurement with minimal restraint. Importantly, lactate values should be interpreted alongside routine physical examination findings, including respiratory rate, heart rate, and presenting complaints, rather than as a replacement for standard clinical assessment. This approach facilitates the integration of lactate measurement into real-time decision-making during episodes of acute decompensation. Consequently, handheld lactate measurement via the paw pad represents a practical option for monitoring high-risk dogs with MMVD, particularly when conventional venipuncture may be poorly tolerated, effectively bridging the gap between the need for urgent metabolic assessment and the risks associated with patient handling.

Several limitations of this study should be acknowledged. First, the retrospective design and relatively small number of dogs in the acute decompensation group limit the strength of conclusions regarding prognostic significance and the generalizability of the proposed lactate threshold. Second, lactate measurements were obtained at a single time point, and serial lactate trends, which may provide additional prognostic insight, were not evaluated. In addition, concurrent hemodynamic or perfusion-related parameters were not assessed, precluding definitive conclusions regarding the underlying pathophysiological mechanisms of hyperlactatemia in this population.

Future prospective studies incorporating larger cohorts, standardized treatment protocols, and serial lactate measurements, alongside concurrent assessment of hemodynamic and perfusion variables, may further clarify the role of point-of-care lactate monitoring in the management and prognostication of dogs with MMVD.

## 5 Conclusion

In conclusion, this study demonstrates that point-of-care whole blood lactate measurement provides clinically relevant information regarding hemodynamic status in dogs with MMVD. Lactate concentrations were not uniformly elevated with disease progression but differed according to clinical status, remaining within the normal range in stage C with controlled CHF and increasing markedly in stage C with acute decompensation CHF. In this context, a lactate value around 3.0 mmol/L was useful for distinguishing dogs without acute hemodynamic compromise from those requiring closer clinical reassessment. These findings support the role of point-of-care lactate measurement as an adjunctive tool for real-time clinical evaluation in dogs with advanced MMVD.

## Data Availability

The original contributions presented in the study are included in the article/Supplementary material, further inquiries can be directed to the corresponding author.
